# Energetic differences between non-domain-swapped and domain-swapped chain connectivities in the K2P potassium channel TRAAK†

**DOI:** 10.1039/c8ra04159h

**Published:** 2018-07-25

**Authors:** Carlos Navarro-Retamal, Julio Caballero

**Affiliations:** Centro de Bioinformática y Simulación Molecular, Facultad de Ingeniería, Universidad de Talca 2 Norte 685, Casilla 721 Talca Chile jcaballero@utalca.cl +56-712-418-850

## Abstract

Two-pore domain (K2P) channels are twofold symmetric K^+^ channels which control cell excitability by enabling the leak of potassium ions from cells in response to physicochemical stimuli. Crystallization of K2P channels revealed the presence of several structural features, which include an external cap. In the available crystallographic structures, the cap is present as non-domain-swapped (NDS) and domain-swapped (DS) chain conformations, where DS chain conformation exchanges two opposing outer helices 180° around the channel. In this work, energy differences between the residues located at the highest point of the cap in NDS and DS conformations were evaluated for TRAAK, a K2P channel that was crystallized in both conformations. Results indicated a preference for DS conformation, but this result is not extensible to TASK K2P channels.

## Abstract 2

In the available crystallographic structures of K2P channels, the cap is present as non-domain-swapped (NDS) and domain-swapped (DS) chain conformations.

## Graphical abstract

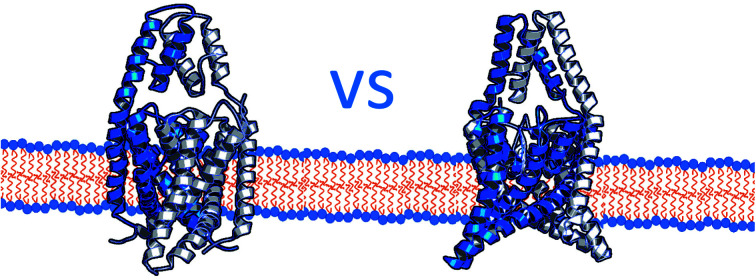

## Introduction

Two-pore domain potassium (K2P) channels are twofold symmetric K^+^ channels formed from two large polypeptide chains.^1,2^ Their function is associated with cell excitability, since they are involved in the maintenance of the resting membrane potential close to the equilibrium potential of K^+^.^3,4^ K2P channels have been identified in mammals, yeast, plants, *etc.*^5^

Typically, K^+^ channel structures are fourfold symmetric tetramers, but K2P channels have a distinct structure since they encode two nonidentical repeats in the large polypeptide corresponding to two identical monomers (denoted as chains A and B in this manuscript) forming a fourfold symmetric channel. K2P channels from mammals contain a large extracellular motif, the so-called cap structure, which extends from the M1 domain to the first pore loop (P1).^1,2^ The role of the cap on functional properties of K2P channels is under investigation.^6^

**Fig. 1:**
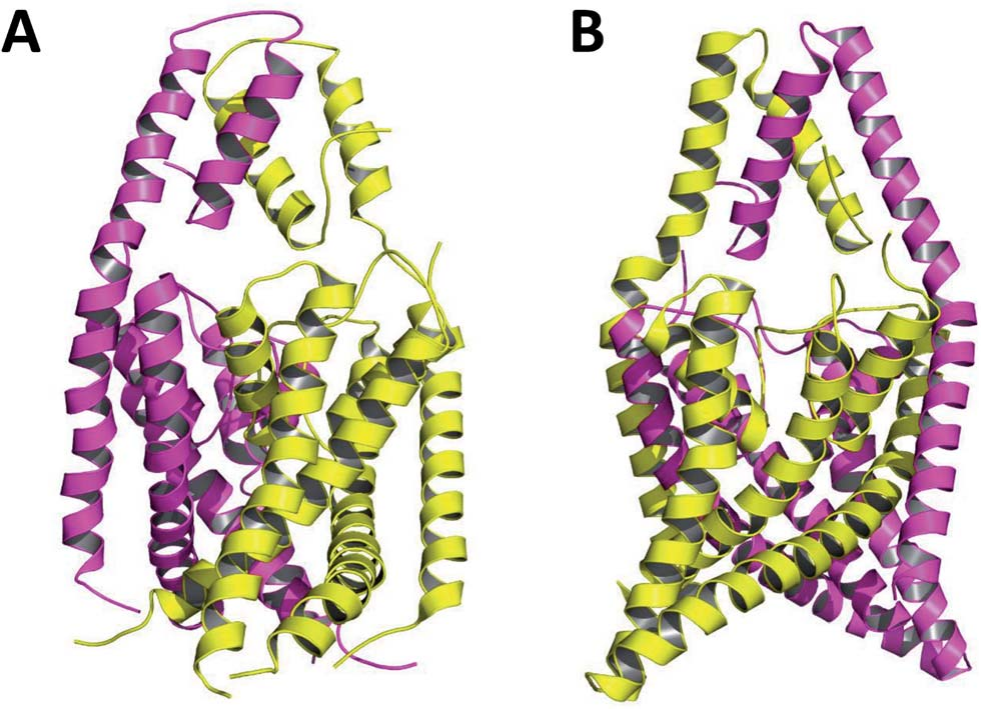
TRAAK in NDS (A) and DS (B) conformations. Chains A and B are represented in purple and yellow respectively.

Crystallized K2P channels structures revealed that the cap contains two helices per monomer denoted as inner and outer helices (Fig. 1). In the first crystallized K2P channels, TWIK-1 and TRAAK, the cap outer helix from each monomer interacts with the inner helix from the same monomer, denoted as non-domain-swapped (NDS) conformation (Fig. 1A).^1,2^ However, in other crystallized K2P channels, TRAAK, TREK1 and TREK2, the cap outer helix from each monomer interacts with the inner helix from the other monomer, denoted as domain-swapped (DS) conformation (Fig. 1B).^7–9^

**Fig. 2:**
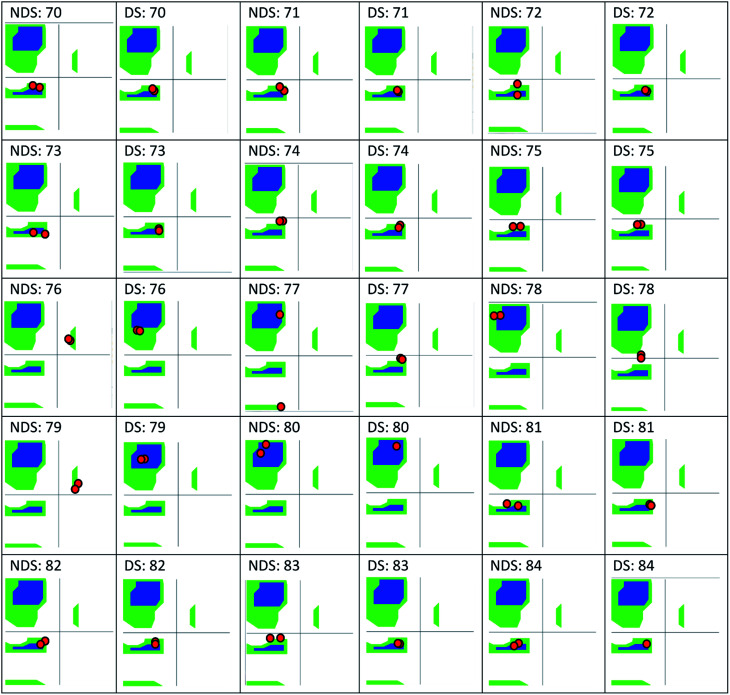
Ramachandran plots for the residues 70–84 of TRAAK in NDS and DS conformations, obtained from crystallographic structures with PDB codes 3um7 (NDS) and 4i9w (DS).

**Fig. 3:**
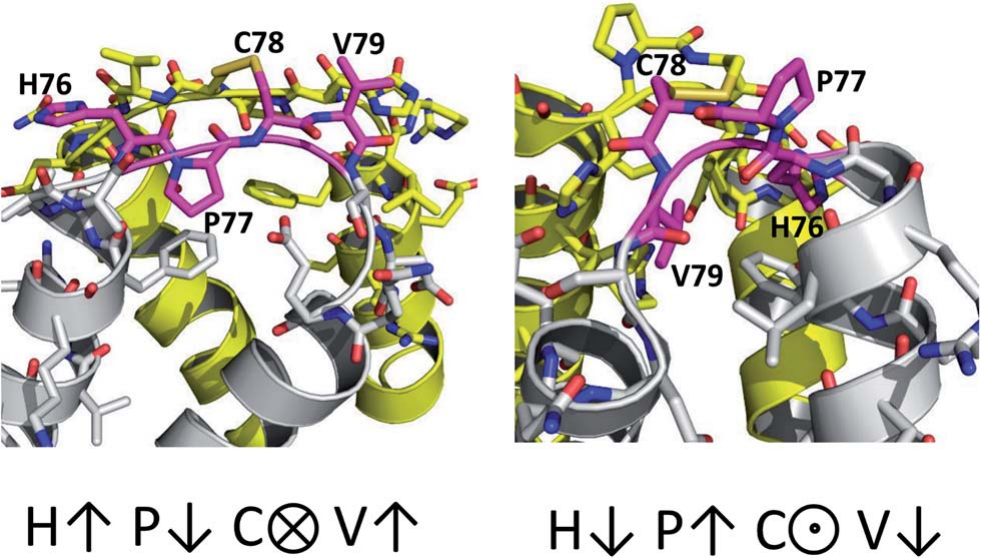
Structural differences between the four residues His76, Pro77, Cys78, and Val79 in NDS (left) and DS (right) conformations.

The only K2P channel crystallized in NDS and DS conformations is TRAAK (protein data bank PDB codes 3um7 and 4i9w, respectively). In general, these structures are very similar, but they differ at the top of the helical cap, where four amino acids (His76. Pro77, Cys78, and Val79) change significantly their orientations in the three-dimensional (3D) space. The residue Cys78 forms the disulfide bond bridging the top of the cap by favoring the two possibilities: NDS or DS. Ramachandran plots for the TRAAK residues from 70 to 84 in NDS and DS conformations (Fig. 2) show that major differences in the backbone between both conformations are located in the four amino acids His76, Pro77, Cys78, and Val79. The structural differences between these residues in NDS and DS conformations are represented in Fig. 3. In NDS conformation, side chains of His76 and Val79 are oriented upwards, and Pro77 is oriented downwards (Fig. 3A); meanwhile, in DS conformation, side chains of His76 and Val79 are oriented downwards, and Pro77 is oriented upwards (Fig. 3B). On the other hand, the disulfide bonds, essential for stability of the cap in TRAAK, are formed between the two Cys78 residues from chains A and B adopting two different orientations. When His76–Val79 are represented from left to right, the disulfide bond points into the plane in NDS conformation; while the disulfide bond points out of the plane in DS conformation (Fig. 3).

The presence of NDS and DS conformations is intriguing, as well as their possible functional significance. Brohawn *et al.* have been proposed that the diffraction data at 3.8 Å for the NDS TRAAK model do not distinguish DS from NDS conformations.^8^ DS structures have been identified thereafter with a better resolution; however, the presence of NDS structures is not discarded. Considering the coexistence of NDS and DS conformations, it would be of interest to know which fraction of the population is DS and what is the driving force behind the DS. Since the positions of atoms that make up the outer and inner helices are the same in NDS and DS conformations, the configuration adopted by residues 76–79 must drive the DS.

In this work, a protocol combining MD and *in silico* alanine scanning mutagenesis free energy analysis was used to evaluate which of the two conformations, NDS or DS, are more favored considering the energetic difference between the four amino acids (His76, Pro77, Cys78, and Val79) at the top of the helical cap and residues at their structural environment. The chemical interactions of the side chains of these residues with other residues at the cap are analyzed, which can provide information about the preponderance of NDS or DS conformation in K2P channels.

## Materials and methods

### Preparation of initial structures and MD simulations

The crystallographic structures of NDS and DS TRAAK were obtained from the Protein Data Bank (PDB codes: 3um7 and 4i9w, respectively). All missing protein hydrogens were added, and protonation states at a neutral pH were assigned for all ionizable residues. Missing residues/atoms were added by using the Prime module included in the Schrodinger suite (Prime, version 3.9, Schrodinger, LLC, New York, NY, 2015). The Protein Preparation Wizard module was used for assigning bond orders and for adding hydrogens to the protein residues (using PROPKA 3.0 to set up the protonation states of the different residues at pH 7).

Proteins were embedded in a 1-palmitoyl-2-oleoyl-*sn*-glycero-3-phosphocholine (POPC) lipid bilayer hydrated on each side. Counterions were added to neutralize the charge of the system, as well as a saline concentration of 150 mM of KCl. The SPC water model was used with the OPLS2005 force field,^10^ and periodic conditions were established.

Desmond module was used to perform all of the molecular dynamics (MD) simulations.^11,12^ Prior the equilibration runs, the default minimization and pre-equilibrated protocol of Desmond was used, which included several short MD runs at different conditions. In order to equilibrate the membrane surrounded the proteins, the “membrane relaxing protocol” module from Desmond was used. This protocol includes 2 runs with 2000 steps of energy minimizations, followed by 60 ps steps of heating (annealing) from 0 to 300 K and 1.1 ns MD by fixing initially all heavy atoms of the system (protein, water and lipids). During the simulation the restraints of water and lipids were decreased allowing their re-accommodation. After removal of non-proper interactions between protein, membrane and water molecules, the default equilibration protocol of Desmond was used to relax the whole system. This protocol includes 5 short MD runs: (i) 100 ps Brownian dynamics (*T* = 10 K, 50 kcal mol^−1^ A^−2^ restraints on heavy atoms, timestep = 1 fs, NVT), (ii) 12 ps MD (*T* = 10 K, 50 kcal mol^−1^ A^−2^ restraints on heavy atoms, timestep = 1 fs, NVT); (iii) 12 ps MD (*T* = 10 K, 50 kcal mol^−1^ A^−2^ restraints on heavy atoms, timestep = 2 fs, NPT), (iv) 12 ps MD (*T* = 10 K, 50 kcal mol^−1^ A^−2^ restraints on heavy atoms, timestep = 2 fs, NPT), and (v) 24 ps MD (*T* = 10 K, no restraints, timestep = 2 fs, NPT). For each system, 50 ns equilibration MD simulations were performed (with a restraint of 0.5 kcal mol^−1^ onto the backbone of the proteins). After this, 150 ns production MD simulations were performed on both systems, where a restraint of 0.25 kcal mol^−1^ was applied onto the protein backbone to maintain both NDS and DS configurations stable. For all the MD simulations a time-step of 2 fs was used, where van der Waals and electrostatic cutoff were set up to 9 Å. The temperature was maintained at 300 K by Nose–Hoover chain thermostat method while the pressure was maintained at 1 bar by Parrinello–Rahman barostat (NPT ensemble). In all simulations the Particle-Mesh Ewald (PME) method was used to estimate coulombic interactions with a cutoff of 9 Å. Coordinates were saved each 25 ps and energies were saved 1.2 ps. Simulations were done by duplicate.

### *In silico* alanine scanning mutagenesis and free energy analysis

To determine the stability of both NDS and DS domain configurations, *in silico* alanine scanning mutagenesis and free energy analysis were performed.^13,14^ To achieve this, two replies of 150 equally spaced snapshots were extracted from the 150 ns MD trajectories. The TRAAK residues from 67 to 84 (except Cys78) were *in silico* mutated by alanine residues and affinity changes due to single mutations were calculated using the Prime Molecular Mechanics-Generalized Born Surface Area (MM-GBSA)^15,16^ in BioLuminate module^14^ of the Schrodinger suite (BioLuminate, Schrödinger, LLC, New York, NY, 2015). After calculating free energies as result of alanine mutation of each residue for NDS and DS conformations, a residue-by-residue comparison was established. The difference Δ*E* (NDS–DS) between energy values due to alanine mutation for each residue represents the amount of free energy in which the residue contributes to favor or disfavor the total free energy of NDS or DS conformation.

## Results and discussion

### Alanine scanning mutagenesis results

Equilibrium and production MD trajectories were analyzed by using potential energy and root mean square deviation (RMSD) plots to guarantee that systems were stable (quality data and RMSD plots are in the ESI, Fig. S1 and S2†). During the MD simulations, the structure of the cap was stable when comparing with TRAAK NDS and DS crystallographic structures. Root mean square fluctuations (RMSF) per residues are reported in the ESI (Fig. S3†), where it is possible to observe that residues at the loop after the cap inner helix have the major fluctuations for NDS and DS conformations. However, both cap outer and inner helices have minor fluctuations, which reflects the stability of the caps. Stability of the secondary structures per residue from the 150 ns production MD simulations for NDS and DS were also analyzed. The analysis for the residues of the cap is reported in the ESI, Fig. S4 and S5;† confirming the stability of the outer and inner helices (before and after the residues at the top of the cap) in NDS and DS TRAAK conformations.

The snapshots were extracted from the 150 ns production MD trajectories for performing *in silico* mutations by alanine of the residues from 67 to 84 without considering Cys78 because this residue forms the disulfide bridge that connects chains A and B. The effects of alanine mutation of each residue on the free energy were evaluated for NDS and DS conformations. These values were compared considering two replies and that each conformation contains each residue at chains A and B. The calculated Δ*E* values comparing each residue in chain A of the NDS conformation with chains A and B of the DS conformation and chain B of the NDS conformation with chains A and B of the DS conformation are reported in the Table 1 for the two replies. An averaged Δ*E* (NDS–DS) value was determined for each residue considering all possible comparisons.

**Table 1:** Free energy differences Δ*E* between residues at the top of the cap of TRAAK for NDS (PDB code: 3um7) and DS (PDB code: 4i9w) conformations calculated from *in silico* alanine scanning mutagenesis

Residue	Δ*E* (NDS–DS) Reply 1^a^ (kcal mol^−1^)				Δ*E* (NDS–DS) Reply 2^a^ (kcal mol^−1^)				Averaged Δ*E* (NDS–DS)^b^ (kcal mol^−1^)	SD^c^
	A3um7-A4i9w	A3um7-B4i9w	B3um7-B4i9w	B3um7-A4i9w	A3um7-A4i9w	A3um7-B4i9w	B3um7-B4i9w	B3um7-A4i9w		
E67*	3.16	1.73	2.70	4.13	3.20	1.77	2.66	4.09	2.93*	0.91
V68	−0.36	−0.61	0.09	0.35	−0.36	−0.61	0.10	0.35	−0.13	0.40
**R69**	**−1.57**	**−1.56**	**−3.86**	**−3.87**	**−1.56**	**−1.55**	**−3.87**	**−3.88**	**−2.71**	**1.24**
E70	−2.48	−3.15	0.83	1.51	−2.49	−3.16	0.70	1.37	−0.86	2.13
K71	2.02	−0.51	0.05	2.58	1.97	−0.56	−0.02	2.51	1.00	1.39
**F72**	**−7.28**	**−7.38**	**−6.29**	**−6.18**	**−7.25**	**−7.36**	**−6.27**	**−6.16**	**−6.77**	**0.59**
**L73**	**−3.69**	**−3.23**	**−2.90**	**−3.36**	**−3.70**	**−3.24**	**−2.94**	**−3.40**	**−3.31**	**0.30**
R74	3.26	1.00	−0.64	1.62	3.21	0.95	−0.62	1.64	1.30	1.48
A75	0.00	0.00	0.00	0.00	−0.38	−0.28	0.18	0.08	−0.05	0.18
**H76**	**−10.94**	**−11.33**	**−15.56**	**−15.16**	**−10.95**	**−11.35**	**−15.57**	**−15.18**	**−13.25**	**2.27**
P77*	4.05	3.87	3.59	3.77	4.03	3.85	3.60	3.78	3.82*	0.17
**V79**	**−13.19**	**−12.90**	**−13.55**	**−13.84**	**−13.20**	**−12.91**	**−13.54**	**−13.83**	**−13.37**	**0.37**
S80	1.98	2.48	0.77	0.27	1.98	2.47	0.76	0.26	1.37	0.95
D81	−1.32	−1.30	−2.51	−2.53	−1.33	−1.31	−2.47	−2.49	−1.90	0.63
Q82	−0.21	−1.58	−0.94	0.43	−0.23	−1.59	−0.93	0.44	−0.58	0.81
**E83**	**−4.95**	**−5.13**	**−2.41**	**−2.23**	**−4.93**	**−5.11**	**−2.47**	**−2.29**	**−3.69**	**1.44**
**L84**	**−2.95**	**−4.17**	**−3.98**	**−2.76**	**−2.96**	**−4.18**	**−3.96**	**−2.74**	**−3.46**	**0.66**

a Δ*E* between the residue at NDS chain A and DS chain A, NDS chain A and DS chain B, NDS chain B and DS chain B, and NDS chain B and DS chain A were considered for each reply. More negative Δ*E* values are in bold letters, more positive Δ*E* values are marked with *.

b Averaged Δ*E* (NDS–DS) was calculated by considering four cases for each reply.

c SD is the standard deviation.

In the Table 1, the residues with negative averaged Δ*E* (NDS–DS) values form more favorable interactions in the DS conformation of TRAAK. Conversely, the residues with positive averaged Δ*E* (NDS–DS) values form more favorable interactions in the NDS conformation of TRAAK. The residues with more favorable interactions in the DS conformation are H76, V79, F72, E83, L73, L84, and R69. On the other hand, the residues with more favorable interactions in the NDS conformation are P77 and E67. It is noteworthy that there are more favorable interactions in the DS conformation of TRAAK due to favorable interactions of more residues with higher Δ*E* (NDS–DS) values. This result suggests that TRAAK DS conformation is energetically more favorable.

**Fig. 4:**
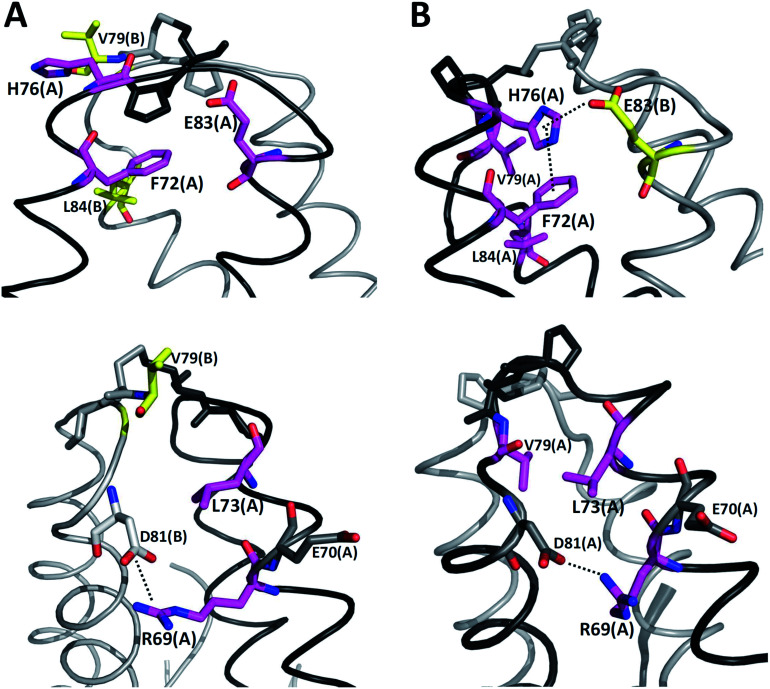
Three dimensional positions of the residues with more negative averaged Δ*E* (NDS–DS) in NDS (A) and DS (B) conformations. F72, H76, E83, V79 and L84 are represented in sticks at top. L73, R69, V79, E70 and D81 are represented in sticks at bottom. Chains A and B are represented in black and gray, respectively. Meanwhile, stick representations in chains A and B are represented in purple and yellow, respectively.

**Fig. 5:**
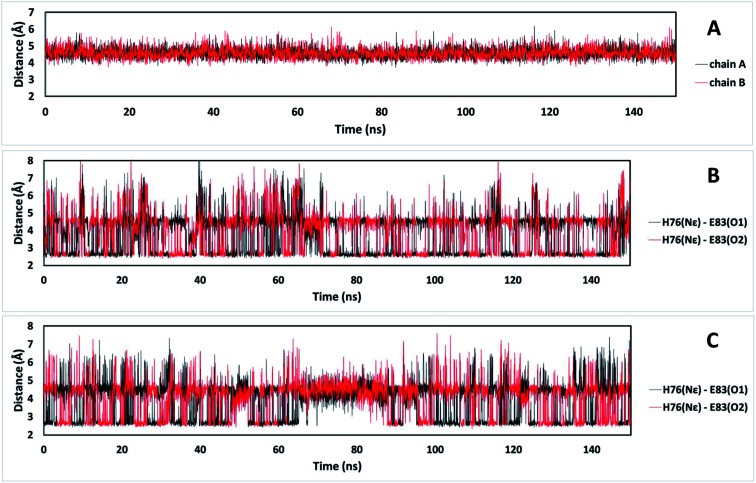
Distances that represent the stability of the triad F72-H76-E83 in the TRAAK DS conformation extracted from 150 ns MD simulation. (A) Distances between the center of mass of the phenyl group of F72 and the center of mass of the imidazole of H76 from the same chain. (B) Distances between the Nε of the H76 from chain A and the carboxylic oxygens (O1 and O2) of the E83 from the chain B. (C) Distances between the Nε of the H76 from the chain B and the carboxylic oxygens (O1 and O2) of the E83 from the chain A.

It is possible to analyze which chemical interactions are established in the DS conformation that contribute to a more favorable energy of association between the interacting residues. Fig. 4 shows that proximities between the identified residues differ between NDS and DS conformations. Interestingly, the residue H76 from chain A, which is protonated, forms a salt bridge with the residue E83 from chain B, and a π-cation interaction with the residue F72 from chain B (Fig. 4B top) when DS conformation is formed. This triad, established between F72, H76 and E83 in TRAAK DS conformation is not present in the NDS conformation (Fig. 4A top). From the MD trajectory of the TRAAK DS conformation (Fig. 5), it is evident that this triad is stable. In all of the 150 ns simulation, the distances between the centers of mass of the phenyl group of F72 and the imidazole of H76 from the same chain (A or B) were stable (Fig. 5A), which indicates that π-cation interaction between residues F72 and H76 are stable in the TRAAK DS conformation. In addition, the analysis of the distances between the Nε of the H76 from chain A or B and the carboxylic oxygens (O1 and O2) of the E83 from the complementary chain indicates that stable hydrogen bonds (HBs) are established between H76 and E83 in TRAAK DS conformation (Fig. 5B and C). These interactions are also electrostatic since both residues have opposite charges (H76 is protonated). The change in the position of H76 in TRAAK NDS conformation prevents the formation of these interactions (Fig. 4A top).

The residue V79 has also a more favorable interaction in the DS conformation. This residue forms hydrophobic interactions with F72, L84 (Fig. 4B top), and L73 (Fig. 4B bottom) from the same chain in the TRAAK DS conformation (defined as the hydrophobic tetrad F72-L73-V79-L84), but the same residue is oriented towards the solvent in the TRAAK NDS conformation (Fig. 4A).

**Fig. 6:**
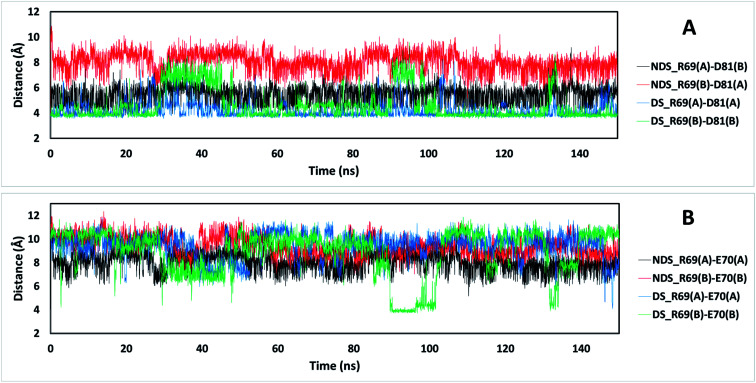
Distances between the guanidine group of residue R69 and carboxylic groups of residues D81 (A) and E70 (B) for TRAAK NDS and DS conformations extracted from 150 ns MD simulation. The black line represents the distances between the C atom of the guanidine of R69 from chain A of the NDS conformation and the C atom of the aspartate carboxylate of D81 from chain B (A) or E70 from chain A (B). The red line represents the distances between the C atom of the guanidine of R69 from chain B of the NDS conformation and the C atom of the aspartate carboxylate of D81 from chain A (A) or E70 from chain B (B). The blue line represents the distances between the C atom of the guanidine of R69 from chain A of the DS conformation and the C atom of the aspartate carboxylate of D81 from chain A (A) or E70 from chain A (B). The green line represents the distances between the C atom of the guanidine of R69 from chain B of the DS conformation and the C atom of the aspartate carboxylate of D81 from chain B (A) or E70 from chain B (B).

The residue R69 was also identified with more favorable interactions in the DS conformation. In the Fig. 4, it is possible to observe that the residues D81 and E70 are close to R69 for both TRAAK NDS and DS conformations with possibilities for establishing electrostatic interactions. In all of the 150 ns simulations, the distances between the R69 side-chain guanidine group and carboxylate groups of D81 and E70 were analyzed for both conformations (Fig. 6). It is possible to see that D81 establishes HB interactions with the guanidine group of R69 from the same chain in the TRAAK DS conformation (Fig. 6A), but the distances between R69 guanidine and D81 carboxylate groups are higher in the NDS conformation. This means that R69 can form more stable electrostatic interactions with D81 in the DS conformation. On the other hand, distances between R69 guanidine and E70 carboxylate groups indicate that these residues do not establish HB interactions for both TRAAK NDS and DS conformations (Fig. 6B). Although, E70 could establish occasional HB interaction with the guanidine group of R69 in the DS conformation.

### NDS or DS conformation in other K2P channels

**Fig. 7:**
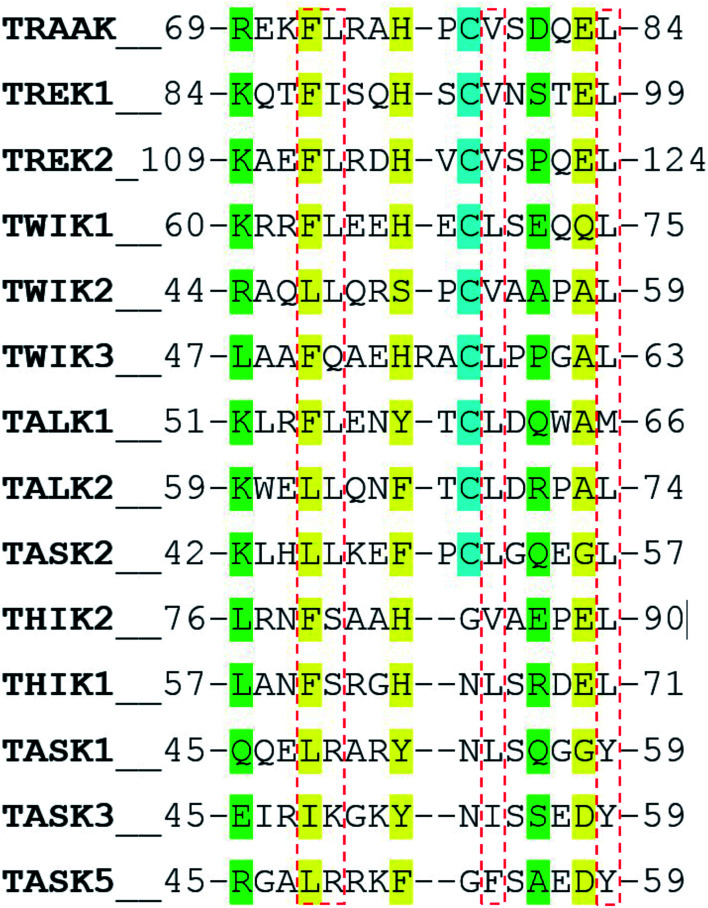
Alignment of the human K2P channels using residues 69–84 from TRAAK as reference. Residues aligned to the triad F72-H76-E83 are colored in yellow. Residues aligned to R69 and E83 are colored in green. Cysteine residues aligned to C78 are colored in cyan. Residues aligned to the hydrophobic tetrad F72-L73-V79-L84 are inside red boxes.

In a recent work, Masetti *et al.*^17^ investigated the TRAAK structure and dynamics by performing MD simulations using both the NDS and the DS conformations as starting conditions. In agreement with our results, they found that channel structures belonging to the NDS topology are less stable than the DS ones, especially at the cap regions. Our results indicate that the TRAAK DS conformation is energetically favored mainly due to the presence of the triad F72-H76-E83 and the hydrophobic tetrad F72-L73-V79-L84 at the top of the cap. A question comes from this analysis: are these features present in other K2P channels? The presence of the triad F72-H76-E83 in other K2P channels could induce to predict that DS conformation is more favored for these K2P channels. An alignment of the human K2P channels using residues 69–84 from TRAAK as reference is presented in Fig. 7 based on the alignment reported in the ref. 2. The triad F72-H76-E83 is conserved in TREK1, TREK2, THIK2, and THIK1. There are available crystallographic structures of TREK1 and TREK2 in the DS conformation (PDB codes: 4twk, 5vkn, 4bw5, 4xdj, *etc.*)^7^ where interactions described in this manuscript are present. In TWIK1, which has been crystallized in NDS conformation,^1^ the member of the triad E83 is replaced by a glutamine (Fig. 7). This replacement could weaken the interactions of the triad since the salt bridge between the histidine and the glutamate of the triad is not present, however the histidine (H67 in TWIK1) and the glutamine (Q74 in TWIK1) could establish an HB to establish the DS conformation. In TWIK3, the member of the triad E83 is replaced by an alanine; this replacement prevents formation of a salt bridge or HB. In other K2P channels, one member or no members of the triad identified in TRAAK are present. In these cases, prevalence of the DS conformation cannot be guaranteed.

The presence of the hydrophobic tetrad F72-L73-V79-L84 in other K2P channels or replacement of these residues by other non-polar residues could also induce to predict that DS conformation is more favored for these K2P channels. The TRAAK tetrad F72-L73-V79-L84 is conserved only in TREK2, and replacements of the tetrad residues by other non-polar residues are observed in the majority of the K2P channels. However, the residue L73 is replaced by neutral polar residues in TWIK3, THIK1, and THIK2 (glutamine and serine) and by positively charged polar residues in TASK1, TASK3 and TASK5 (arginine and lysine). This analysis suggests that the hydrophobic tetrad is not formed in these K2P channels; therefore, the hydrophobic packing observed in the DS conformation of TRAAK is not present when the DS conformations of these K2P channel are formed. Once again, prevalence of the DS conformation cannot be guaranteed in these cases.

Finally, the alignment in Fig. 7 shows that the pair of residues R69-D81 are not conserved in K2P channels.

In the last years, there is a special interest in the modeling of TASK channels TASK1, TASK2 and TASK3.^18–20^ These channels do not contain any member of the triad F72-H76-E83 and at the top of the cap. Additionally, TASK1 and TASK3 do not contain a cysteine connecting chains A and B and have a positively charged residue instead the TRAAK residue L73 of the hydrophobic tetrad. According to our evidences and the available structural data, it is not supported that DS conformation be prevalent in these channels.

### The existence of the NDS conformation in K2P channels

The existence of the NDS conformation in K2P channels has been questioned. Brohawn *et al.*^8^ suggested that the first model of TRAAK is not in a NDS conformation because the low resolution of the diffraction data for the original model reported in the ref. 2 does not distinguish DS from NDS models. However, there is no demonstration that NDS is not a conformation of K2P channels. In fact, scientists use NDS conformation to study TWIK-1,^21–23^ which was crystallized in the NDS conformation also with a low resolution.^1^ The presence of the DS conformation in TWIK-1 has not been demonstrated; therefore, it makes sense that scientific community considers that TWIK-1 adopts the NDS conformation, considering the structural information until now. Authors who have conducted scientific studies on TASK K2P channels have also been considered that these channels are in the NDS conformation,^18,19,24^ because it is easier to establish interactions in the NDS conformation when two independently K2P folded subunits assemble. The formation of the DS conformation requires a large movement of the outer helices of the cap,^8^ and it is not demonstrated that this process occurs in TASK channels.

Our results could be interpreted as a support of the no existence of TRAAK in the NDS conformation, since the presented energetics in Table 1 suggest a massive preference (around 10 kcal mol^−1^ for several residues) for the DS conformation, supporting the suggestion of Brohawn *et al.* in the ref. 8. However, it is important to consider that our calculations are local (only a few residues were compared) and MM/GBSA is not accurate when calculating absolute free energy values; *i.e.* MM/GBSA free energy calculations do not give quantitative estimates for the binding free energies.^16,25–33^ It is wise to consider Prime MM/GBSA energy values for comparisons, considering relative free energy values. Therefore, our work could indicate a high prevalence of the DS conformation in TRAAK, which cannot be extended to other K2P channels according to the sequence analysis.

## Conclusions

Crystallized K2P channels revealed the presence of an external cap adopting NDS or DS conformations. After the report of K2P channels structural data since 2012, it is intriguing to know whether most of the functional K2P channels or only a fraction of them are in a DS conformation. TRAAK is the only K2P channel crystallized in NDS and DS conformations. Our protocol, combining MD and *in silico* alanine scanning mutagenesis free energy analysis, indicated that TRAAK DS conformation is more favored considering the energetic difference between the four amino acids (His76. Pro77, Cys78, and Val79) at the top of the helical cap and residues at their structural environment. The main chemical elements that sustain this statement are numbered below:

- A triad is established between F72, H76 and E83 in TRAAK DS conformation which is not present in the NDS conformation. Salt bridge and HB are established between H76 and E83, and a π-cation interaction is established between H76 and F72 in the DS conformation.

- V79 forms hydrophobic interactions with F72, L84, and L73 in the TRAAK DS conformation, but the same residue is oriented towards the solvent in the TRAAK NDS conformation.

- R69 forms more stable electrostatic interactions with D81 in the DS conformation with a stable HB.

An alignment of the human K2P channels using TRAAK as reference shows that the triad F72-H76-E83 is not conserved in most of the K2P channels, which suggests that available structural data do not support formation of DS conformations in these channels. However, K2P channels that conserve this triad (TREK1, TREK2, THIK2, and THIK1) should have a prevalence of the DS conformation.

## Conflicts of interest

There are no conflicts to declare.

## Supplementary Material

RA-008-C8RA04159H-s001
