# Supplementary material for: Energetic differences between non-domain-swapped and domain-swapped chain connectivities in the K2P potassium channel TRAAK
Source: RSC Adv. 2018 Jul 25;8(47):26610–8. doi: 10.1039/c8ra04159h (PMC9083029; doi:10.1039/c8ra04159h)

Electronic Supplementary Material (ESI) for RSC Advances.
This journal is © The Royal Society of Chemistry 2018

Supplementary material

Fig. S1 Quality data of 50-ns equilibration MD simulations for NDS (at the left) and DS (at the right) TRAAK models. (a) Evolution of the potential energy, (b) evolution of the total volume of the system, (c) evolution of the temperature.

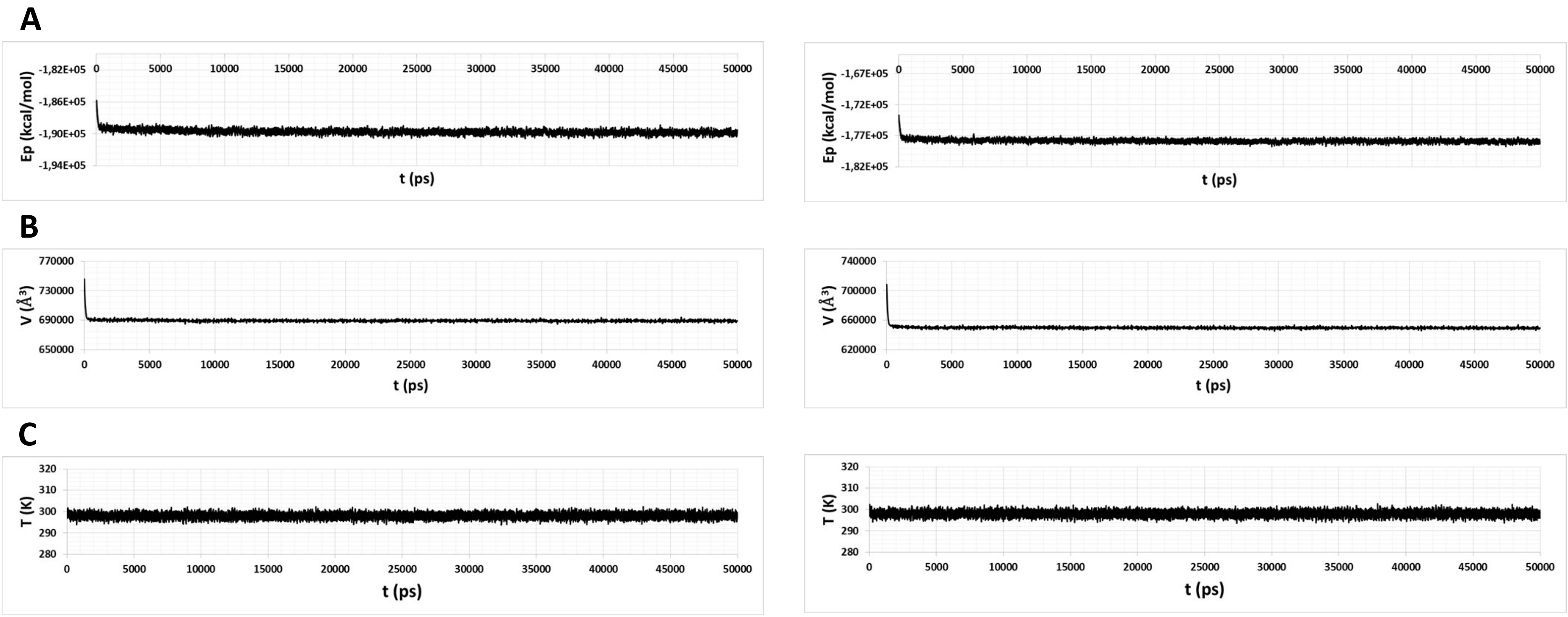

Fig. S2 RMSD values (in Å) of 50-ns equilibration MD (before the red line) and first 100-ns production MD (after the red line) simulations for NDS (at the top) and DS (at the bottom) TRAAK models.

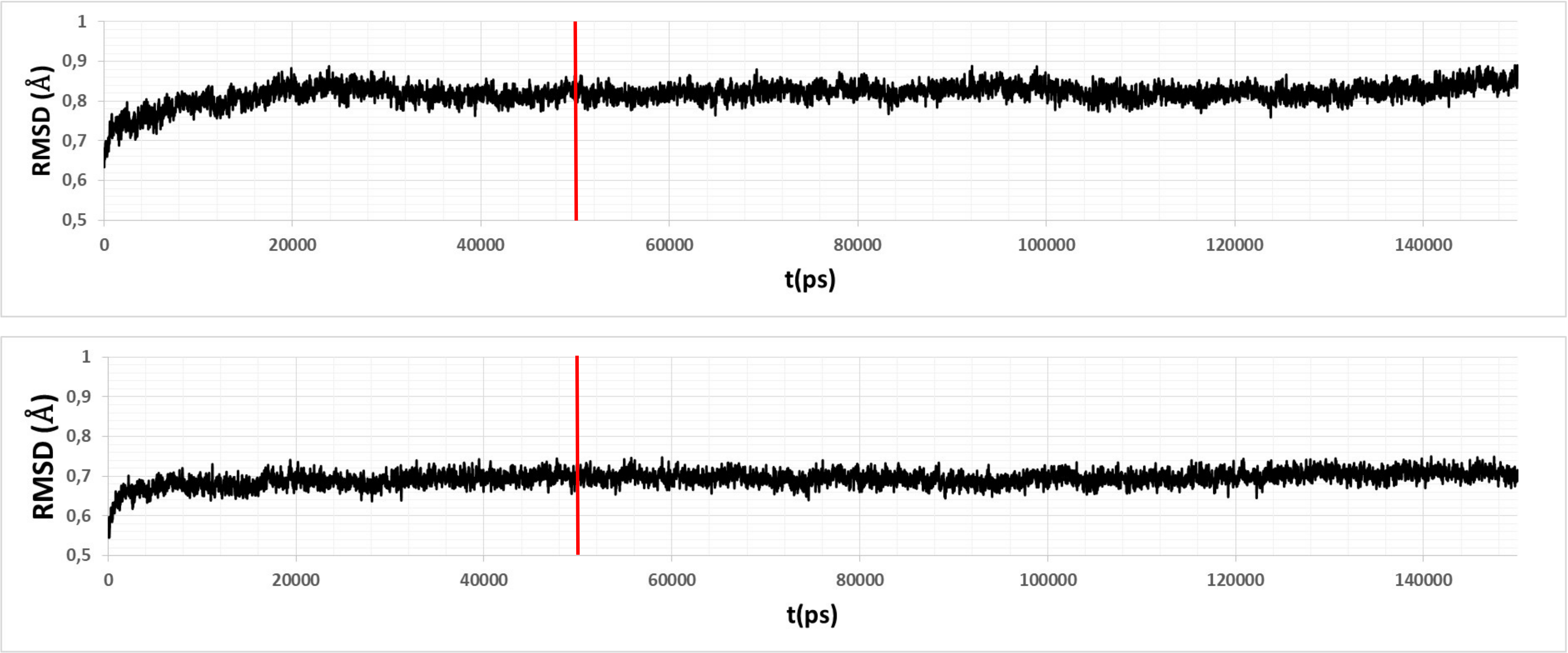

Fig. S3 Root mean square fluctuation (RMSF) values (in Å) per residues from the 150-ns production MD simulations for NDS (chains A and B in black and blue, respectively) and DS (chains A and B in red and orange, respectively) TRAAK models. Cap outer and inner helices are indicated with red and blue lines respectively.

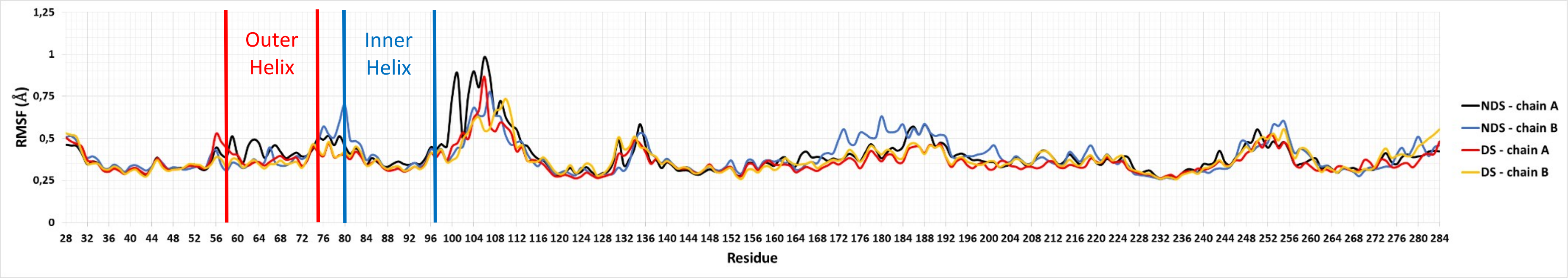

Fig. S4 Stability of the secondary structure per residues of the cap from the 150-ns production MD simulations for NDS (chains A and B at left and right). Helix amino acid conformations are represented in red. Residues at the top of the cap (H76, P77, C78 and V79) are highlighted in yellow.

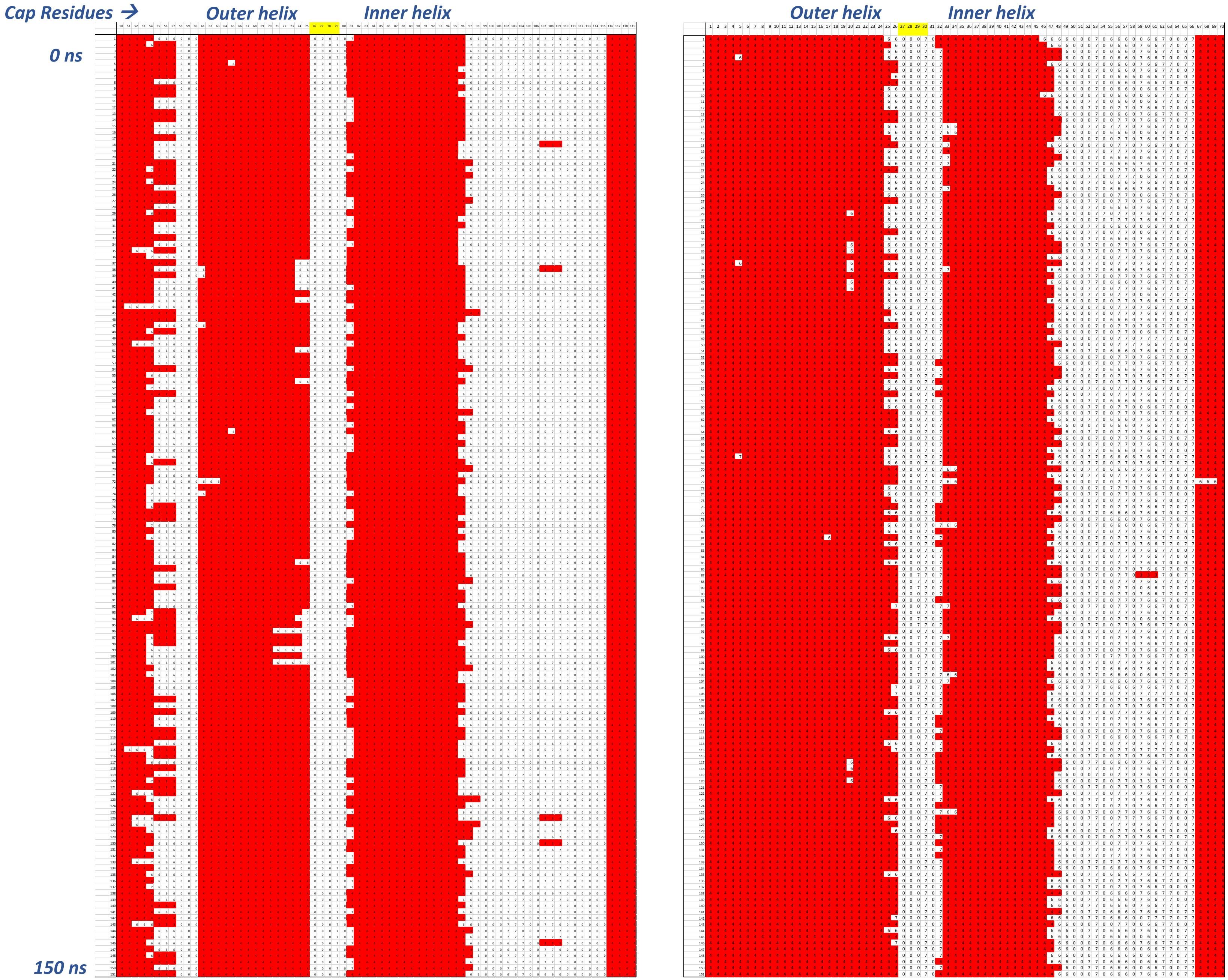

Figure S5. Stability of the secondary structure per residues of the cap from the 150-ns production MD simulations for DS (chains A and B at left and right). Helix amino acid conformations are represented in red. Residues at the top of the cap (H76, P77, C78 and V79) are highlighted in yellow.

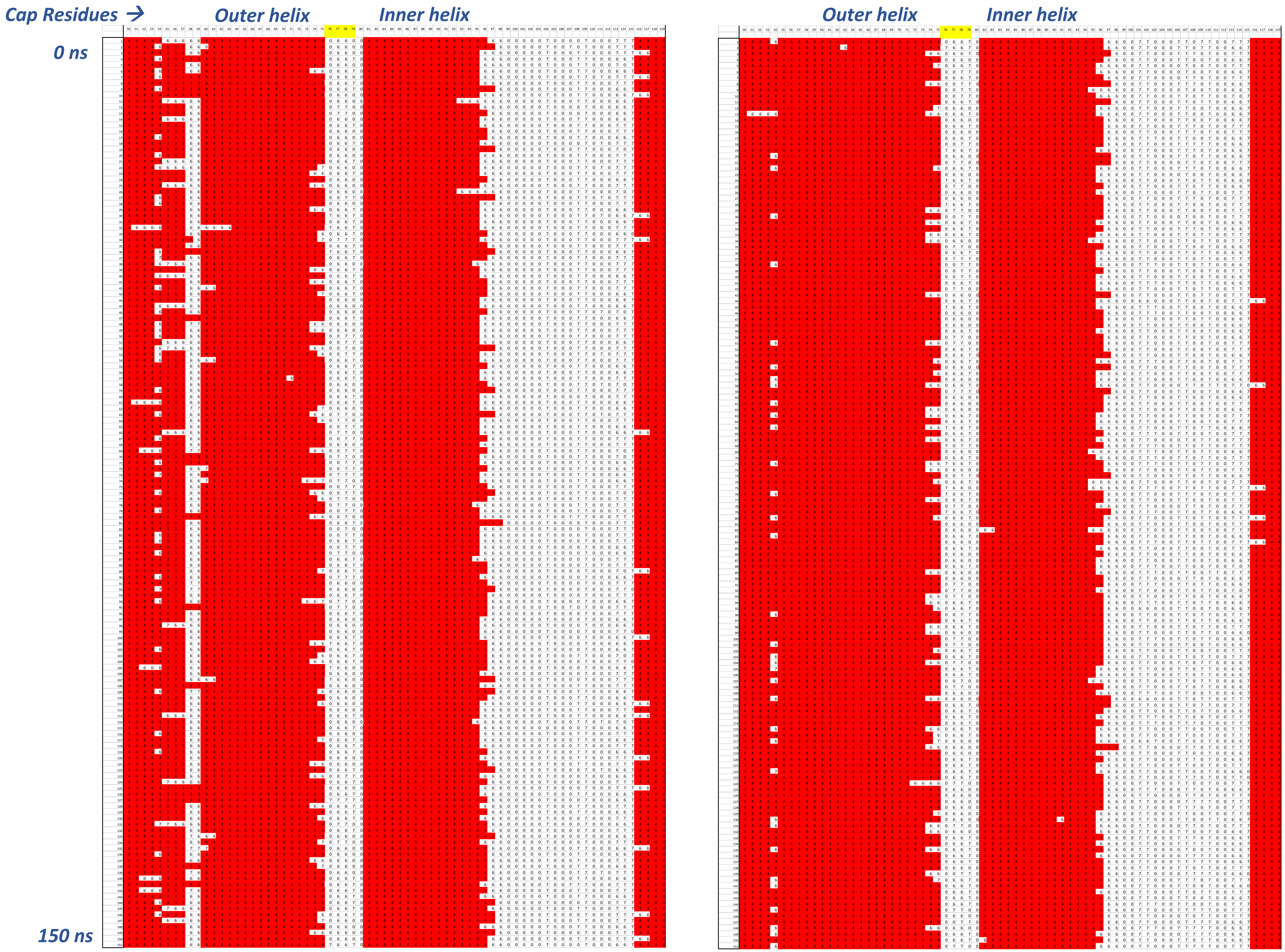

Supplement: RA-008-C8RA04159H-s001 [file RA-008-C8RA04159H-s001.pdf]
